# Interplay of Environmental, Individual and Genetic Factors in Rheumatoid Arthritis Provocation

**DOI:** 10.3390/ijms23158140

**Published:** 2022-07-23

**Authors:** Marina Arleevskaya, Elena Takha, Sergey Petrov, Gevorg Kazarian, Yves Renaudineau, Wesley Brooks, Regina Larionova, Marina Korovina, Anna Valeeva, Eduard Shuralev, Malik Mukminov, Olga Kravtsova, Andrey Novikov

**Affiliations:** 1 Central Research Laboratory, Kazan State Medical Academy, 420012 Kazan, Russia; miwutka@yandex.ru (E.T.); seregapetrov96@yandex.ru (S.P.); gevorg.kazarian@mail.ru (G.K.); reginalarionova1993@mail.ru (R.L.); koporulina.mo@gmail.com (M.K.); anna-valeeva@mail.ru (A.V.); eduard.shuralev@mail.ru (E.S.); malik-bee@mail.ru (M.M.); 2 Institute of Fundamental Medicine and Biology, Kazan (Volga Region) Federal University, 420008 Kazan, Russia; okravz@rambler.ru; 3 Institute of Environmental Sciences, Kazan (Volga Region) Federal University, 420008 Kazan, Russia; 4 Department of Immunology, CHU Toulouse, INSERM U1291, CNRS U5051, University Toulouse IIII, 31000 Toulouse, France; renaudineau.y@chu-toulouse.fr; 5 Department of Chemistry, University of South Florida, Tampa, FL 33620, USA; wesleybrooks@usf.edu; 6 Mathematical Center, Sobolev Instiute of Mathematics, Siberian Branch of Russian Academy of Sciences, 630090 Novosibirsk, Russia; a.hobukob@gmail.com

**Keywords:** rheumatoid arthritis, environmental factors, infection, mental stress, perinatal factors, gender

## Abstract

In this review, we explore systemization of knowledge about the triggering effects of non-genetic factors in pathogenic mechanisms that contribute to the development of rheumatoid arthritis (RA). Possible mechanisms involving environmental and individual factors in RA pathogenesis were analyzed, namely, infections, mental stress, sleep deprivation ecology, age, perinatal and gender factors, eating habits, obesity and smoking. The non-genetic factors modulate basic processes in the body with the impact of these factors being non-specific, but these common challenges may be decisive for advancement of the disease in the predisposed body at risk for RA. The provocation of this particular disease is associated with the presence of congenital loci minoris resistentia. The more frequent non-genetic factors form tangles of interdependent relationships and, thereby, several interdependent external factors hit one vulnerable basic process at once, either provoking or reinforcing each other. Understanding the specific mechanisms by which environmental and individual factors impact an individual under RA risk in the preclinical stages can contribute to early disease diagnosis and, if the factor is modifiable, might be useful for the prevention or delay of its development.

## 1. Introduction

Rheumatoid arthritis (RA) is a recognized model of multifactorial diseases, developing as an inappropriate response to environmental challenges in a genetically predisposed individual. Indeed, less than 30–60% of RA risk is due to the genetic propensity, whereas 40–70% is due to the influence of non-genetic factors [1]. Therefore, the study of the role of non-genetic factors in RA development is of great interest. In addition, in contrast to genetic predisposition, if we have a clear idea of the prognostic significance of these factors, we can manipulate at least some of them in order to prevent or delay RA onset. Even if we cannot eliminate or weaken the effect of any factor, providing clearer understanding of the pathogenic mechanisms of its effect on persons at risk can contribute to the development of approaches to safe therapy by inhibiting undesirable effects in the preclinical RA stages.

In this review, we discuss systematization of the knowledge regarding pathogenic mechanisms that have a triggering effect of non-genetic factors leading to RA development.

## 2. Methods

**Figure 1:**
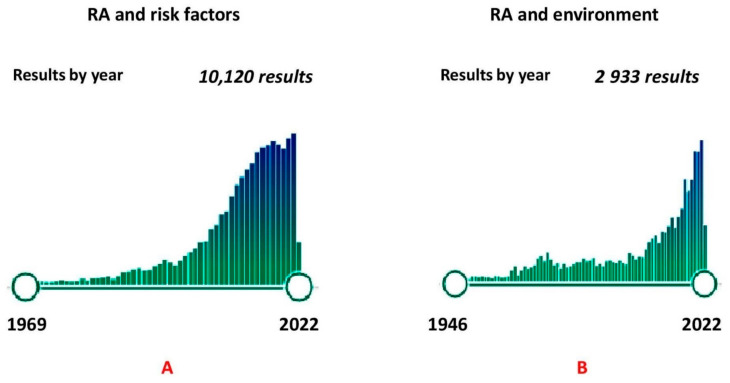
Results of PubMed publications searching with keywords (**A**) “RheumatoidArthritis and risk factors” (10,120 results in 1969–2022), (**B**) “Rheumatoid arthritis andenvironment” (2933 results in 1946–2022).

PubMed publications were selected using the following keyword pairs: “RA and risk factors” (since 1969–2022years–10,120 results, Figure 1) and “RA and environment” (1949–2022years–2933 results, Figure 1), as well as “RA and family aggregation”, “RA and ethnicity”, “RA and socioeconomic indicators”, “RA and perinatal factors”, “Perinatal programming and immune system”, “RA and gender”, “Immune system and sex hormones”, “RA and age”, “Immune system and age”, “RA and body mass index”, “Obesity and inflammation”, “RA and eating habits”, “RA and coffee”, “Caffeine and immune system”, “Alcohol and immune system”, “RA and alcohol”, “RA and smoking”, “RA and pollutants”, “RA and ecologic factors”, “RA and occupational hazards”, “Immune system and ecologic factors”, “RA and mental stress”, “Mental stress and pathogenesis”, “Mental stress and immune system”, “RA and sleep deprivation”, “Sleep deprivation and immune system”, and “RA and infections”.

After analyzing most of the publications, we selected the most informative of them and tried to create a complete picture of the possible relationships of RA and risk factors.

### 2.1. Facts of Genetic and Non-Genetic Factor Interplay

A detailed review of the genetic background of RA is not the subject of this article, but it makes sense to mention some of the particularly pertinent known facts.

#### 2.1.1. RA-Associated HLA-DRB1

Allele variants (Shared epitopes, SE) show significance due to increased density of DR4 and DR1 molecule expression on antigen-presenting cells, leading to noticeable presentation of low-affinity peptides—a process of little importance for the development of a T-cell response at a more typical physiological density of DR molecules [2]. Presentation of low-affinity auto-peptides leads to autoreactive T cell activation.

A pronounced SE association and anti-cyclic citrullinated peptide antibodies (ACCP), but not rheumatoid factor (RF) production, is well documented. In a number of experimental models, the stability of SE complexes (DRB1*01and*04) with citrullinated proteins and other arthritogenic antigens was demonstrated to be increased in comparison with antigens not present in RA pathogenesis. And, in addition, the interaction of these complexes with T-lymphocyte receptors (TCR), as well as their influence on T-lymphocyte proliferation and Th1 cytokine response development, was demonstrably increased [3,4,5,6,7,8].

The affinity of TCR binding to HLA molecule–antigen complexes plays an important role in the lymphocyte maturation: too strong or too weak binding leads to a corresponding removal of clones. Despite their inconsistency, the experimental results demonstrated that interaction of these complexes with TCRs is optimal for the development of an immune response to citrullinated peptides and activation of proinflammatory cytokine synthesis.

The list of non-genetic factors triggering HLA gene expression:

1. Expression is due to appearance of citrullinated peptides in the infectious inflammatory sites [9];
2. Smoking leads to the exposure of citrullinated proteins in the respiratory tract [10];
3. Both SE and aryl hydrocarbon receptor (AhR)—a transcription factor mediating xenobiotic effects of many pollutants (including tobacco)—act as signal transduction ligands facilitating differentiation of Th17 cells and osteoclasts; nuclear factor kappaB-mediated synergistic interaction between the SE and AhR pathways was demonstrated in severe arthritis in mice [11].

#### 2.1.2. Non-HLA Gene Polymorphisms

About 100 RA susceptibility loci were identified with SNP accumulation in T-cell and B-cell pathways, nuclear factor kappa B (NFkappaB) and Jak/STAT-signaling cascades, cytokine signaling pathways, proliferation and/or impaired hematopoietic and immune cells [12,13,14,15]. Collectively, RA-associated non-HLA gene polymorphisms are due to the insufficient inhibition of immune cell activity and to an excessive and long proinflammatory reply to external challenges.

The list of non-genetic factors triggering expression of these genes:

4. Infections due to activation of NFkappaB and Jak/STAT-signaling cascades, cytokine signaling pathways, immune cells proliferation, appearance of citrullinated peptides in the infectious inflammatory site [16,17,18,19,20,21];
5. Smoking due to citrullinated proteins exposure in lungs [22,23];
6. Miscarriage, complicated pregnancy, childbirth leading to a Th1-immune reaction flare-up and proinflammatory cytokine expression [24];
7. Obesity linked to increased proinflammatory cytokine levels [25];
8. Mental stress and sleep deprivation due to increased proinflammatory cytokine levels [26,27,28].

### 2.2. Complex Non-Genetic Factors

#### 2.2.1. Family Aggregation

The obvious components of RA family clustering are genetic risk and shared environmental factors [29]. Heritability of ACCP-positive RA is~50% and of ACCP-negative RA is ~20% [29]; therefore, genetic and non-genetic factors might be of relatively equal importance for seropositive RA development, whereas non-genetic factors might be more important for seronegative RA. Study of the contribution of SE, 76 other gene SNPs and non-genetic factors determined to be shared by the family members (smoking, alcohol intake, parity, silica exposure, BMI, fatty fish consumption, socio-economic status) demonstrated: (1) SE together with 76 SNPs explained about 20% of the familial risk [30] and (2) studied non-genetic risk factors did not explain any significant part of the familial risk in both seropositive and negative RA. Therefore, family history of RA remains an important independent risk factor for RA. Many non-genetic factors besides the ones studied might be due to the RA family clustering with an essential cumulative effect.

The list of triggering and protective family associated factors:

- Genetic;
- Infections;
- Microbiome;
- Lifestyle;
- Ecology (when living together);
- Unknown??

#### 2.2.2. Ethnicity

The overall adult RA prevalence is approximately 0.5%; however, considerable variation exists between ethnicities, with a higher prevalence observed in those of European ancestry (0.3–1.1%) than in those of Asian ancestry (0.1–0.5%) [31,32], and even higher prevalence (approximately 5–7%) has been reported in Native American populations [33]. That is due to the known genetic differences between the populations as well as the obvious but not-well-studied differences in non-genetic factors (lifestyle, eating habits, socio-economical differences and so on).

Some examples of the known ethnic genetic specificity [15,34,35,36]:

HLA-DRB1 SEs: in Europeans—HLADRB*0401, HLA-DRB*0404, HLA-DRB*0101,
in Asians—HLADRB*0405, HLA-DRB*0101, HLA-DRB*0901
PTPN22 SNPs confirmed for Europeans, rare in Asians
TRAF1/C5 confirmed for Europeans, suggested for Asians
STAT4 confirmed both for Europeans and Asians
CD40 confirmed for Europeans, no associations for Asians
CTLA4 confirmed both for Europeans and Asians
PADI4 suggested for Europeans, confirmed for Asians
FCRL3 suggested for Europeans, confirmed for Asians

The list of suggested ethnicity-associated triggering and protecting factors:

- Genetic;
- Lifestyle;
- Eating habits;
- Mentality;
- Socioeconomic indicators;
- Climate.

#### 2.2.3. Socioeconomic Indicators

A lower educational level ≤ 8 years (OR = 2.42, 95% CI 1.18–4.93 vs. University degree) and living in poverty contributed (OR = 2.96, 95% CI 1.88–4.65, *p* < 0.001) to RA development [37,38].

An a priori list of provoking/protective socioeconomic factors:

- Professional activity—lower status—more professional occupational hazards?
- Lifestyle
- Food habits
- Lower status—fewer opportunities to protect/improve one’s health
- Less education—less understanding of the importance of regular examinations
- Living condition quality—overcrowding, uncomfortable housing—infections

### 2.3. Anthropological Indicators

#### 2.3.1. Perinatal and Early Life Factors

Twin studies demonstrated a high sensitivity of the fetal genome to the effects of various factors of maternal origin.

In monozygotic twins sharing a chorionic shell during the intrauterine development, the pattern of gene methylation differed from that in pairs who developed in two separate chorionic shells. In adulthood, this can lead to significant differences in organs and system functioning in these genetically identical individuals [39]. The impact of perinatal factors on the development of cardiovascular diseases, type II diabetes, and obesity has been demonstrated [40,41,42]. The bulk of publications on this issue was devoted to allergic diseases. A correlation ofTh1/Th2/Th17cytokine levels, pro-inflammatory cytokine/chemokine indexes, and IgE levels in peripheral blood of pregnant women at 34 weeks of gestation and in umbilical cord blood of their newborns was demonstrated, persisting one year after delivery [43]. A relationship of cytokine levels in mother and her child and a number of disorders of fetal immune system maturation might be due to epigenetic modeling during prenatal development and abnormal gene expression [44,45]. Such programming of the fetal immune system under conditions of a complicated pregnancy might occur in families with a history of RA. Infections during the first year of life were associated with increased risk of seronegative RA (OR = 2.6). Maternal smoking during pregnancy changes the pattern of newborn gene methylation; the abnormally methylated gene clusters included significant impact on RA pathways related to cell cycle, angiogenesis, T cell regulation and other white blood cell related pathways, which increased the risk for RA development [46,47]. Peculiarities of prenatal development and pregnancy abnormalities were not necessarily closely tied to future RA development and even might be protective. For example, low birth weight (OR = 0.7), being small for the gestational age (OR = 0.5) and preterm birth (OR = 0.6) were shown to have a borderline protective effect [48].

List of hypothetical links of perinatal factors and RA risk in predisposed individuals:

Intrauterine fetal infections ⇒ immune system programming
Maternal infection during pregnancy ⇒ programming of fetal immune system
Complicated pregnancy and childbirth ⇒ fetal immune system programming
Microbiome—maternal origin, breast feeding/bottle feeding
Maternal smoking
Early life infection ⇒ programming the immature immune system and impact formation of the microbiome

#### 2.3.2. Gender Associated Factors 

Sexual dimorphism in expression of human rheumatic diseases involves immunomodulatory effects of post-puberty levels of sex steroid hormones [49]. Due to the presence of hormone receptors on immune cells [50], sex hormones might influence different aspects of immune system functioning and potentially affect the risk, activity and progression of RA [51]. Sex hormones undergo complex dynamics during pregnancy, childbirth, the postpartum period, and the onset of menopause [52,53,54,55].

These multidirectional changes in sex hormones are superimposed on the effects of adrenocorticotropic hormone and cortisol with well-known impacts on the immune system and inflammation. Moreover, the levels of corticosteroid production are different in normal and complicated pregnancies [55]. Low maternal cortisol may influence the fetal hypothalamic–pituitary–adrenal axis (HPA) and disease patterns later in life following a complicated pregnancy [56]. A negative association between maternal cortisol and infant birth weight was demonstrated [57].

We tried to link sex-related events and associated hormonal fluctuations with the impact on the development of RA (Supplementary Table S1).

Repeated normal pregnancies, childbirth, postpartum breastfeeding with normal feedback in the network of sex hormones and glucocorticoids ⇒ bursts of production of these hormones with a protective effect ⇒ reduced RA risk;

Normal pregnancy with hidden feedback impairments in the sex hormone network and glucocorticoids ⇒ RA onset within 1 year after delivery;

Adverse pregnancy as a clinical manifestation of feedback impairments in the network of sex hormones and glucocorticoids ⇒ RA triggering

Menopause ⇒ decrease in hormone levels and their protective effects ⇒ RA risk

#### 2.3.3. Age

RA affects any age group, with the peak occurring during the sixth decade of life [58,59]. The triggering role of age might be due to the decline in host immunity with promotion of immune reactivity to self-antigens, weakened antimicrobial immunity, predisposition for tissue inflammation and osteoarthritis due to chronic microtraumas of joint tissues [60,61].

Impact of age on the immune system and joint tissues [61]:

- Weakened antimicrobial immunity;
- Susceptibility to respiratory infections;
- Reactivation of chronic viral infections;
- Predisposition for tissue inflammation;
- Osteoarthritis due to chronic microtraumas of joint tissues.

#### 2.3.4. Body Mass Index

Obesity is recognized as a chronic low-grade systemic inflammatory state, NFκappaB and NLRP3 inflammasome signaling pathways and proinflammatory cytokine transcription being the key events [25].

Obesity effects on the immune system [62] due to RA triggering: effector/memory T-cell population increase, impoverishment of TCR diversity, M2 to M1 macrophage shift in adipose, increase in the TH1 cell population and decrease in the Treg cell numbers in adipose, NF-kB cascade activation in PBMCs, increased production of MIF, IL-6, TNF-a, MMP-9 mRNA expression in PBMCs, inhibition of phagocytic activity of PBMCs and increased infection susceptibility [62]. Chronic joint tissue microtraumas is additive.

Link of BMI and RA:

- Overweight ⇒ chronic low grade, systemic inflammatory state
- Microbiome structure peculiarities
- Overweight ⇔ stress ⇔ sleep deprivation
- Overweight ⇒ infections
- Overweight ⇒ chronic joint tissue microtraumas

#### 2.3.5. Eating Habits

Diets containing fatty fish (marine omega-3 fatty acid, OA3FA) are beneficial for RA prevention and reduce the need for nonsteroidal anti-inflammatory drugs [63]. RA development is associated with lower levels of OA3FA, especially in ACCP-positive persons at risk. Three mechanisms were described: (I) OA3FA inhibits proinflammatory eicosanoid production (prostaglandinE2 and leukotriene B4), which in turn inhibits NFkappaB activation and proinflammatory interleukin production, ultimately resulting in autoreactive B cells and synoviocyte activation and maturation; (II) OA3FA promotes cell surface receptor expression (vascular cell adhesion molecule (VCAM)-1, and PPARγ in monocytes) or repression (CCL5,HLA-DQ/DR), leading to reduction of Th17 differentiation, enhancement of Foxp3+CD4+T cells regulatory functions, promotion of M2 polarization and (III) interaction between OA3FA and the SE is suspected as an inverse association between OA3FA concentrations, and SE+RF (OR = 0.26), or SE+ACCP (OR = 0.44) was reported in RA risk cohorts. The other promising approach for protection might be the Mediterranean diet with low saturated fat content, contributing to a decrease in RA activity [64]. Though the evidence is insufficient to unconditionally include this diet in the recommendations for RA patients, at least there is reason for more in-depth research, since it was demonstrated that this diet may lower RA severity due to antioxidant and anti-inflammatory properties [65,66].

The earliest preclinical events in RA development were proven to start at the barrier tissue mucosal membranes, including the gut. Although the data on RA association with microbiome features are extremely contradictory, mainly due to small sample sizes, a priori it can be assumed that microbiome–local immunity and barrier cell interactions play a role in the earliest RA stages. So, dietary manipulation of the microbiome might be effective in suppressing undesirable events that take place in early RA stages, when the process has not yet gone awry [67,68,69,70].

On the other hand, a Western diet with higher intake of red and processed meats, sweets, and refined grain associated with elevated inflammatory markers might increase RA risk [71].

### 2.4. Salt and RA

Of interest is the number of publications discussing the problem linking RA to sodium chloride consumption. This problem has not been sufficiently studied with regard to RA, probably due to significant difficulties in technical methods. However, the in vitro effects of excess sodium chloride concentrations on immune cells suggest a potential trigger role in the pathogenesis of RA.

Indeed, salt increased migration of macrophage-like RAW264.7 cells in a dose-dependent fashion with no migratory response noted in isotonic or hypotonic media controls, or other osmo-active agents [72], and high NaCl concentrations promoted IFNβ production and signaling in human and mouse macrophages and inhibited M2 macrophage activation [73,74]. Dendritic cells treated with high NaCl concentrations produced increased levels of interleukin-1β and promoted T cell production of cytokines IL-17A and interferon gamma (IFN-γ) [75].Induction of Th17 response due to the activation of glucocorticoid kinase 1 (SGK1), a serine/threonine kinase, governing Na(+) transport and salt (NaCl) homeostasis in the cells, on the one hand, is critical for regulating IL-23R expression and, on the other hand, for stabilizing the TH17 cell phenotype and controlling the balance between regulatory Treg and Th17 cells [76]. Therefore, salt promotes the suppression of Treg proliferation and function as well [76,77]. So, in vitro high salt concentrations demonstrated a perceptible effect, including triggering Th-17 responses, which is relevant in RA pathogenesis.

Next, experiments in rodent models demonstrated more severe clinical and histological arthritis in the high-salt diet and collagen-induced arthritic mice, together with higher numbers of Th17 cells among splenocytes and increased expression of synovial and intestinal IL-17, compared to control collagen-induced arthritis (CIA) mice fed a normal salt diet [78]. Sehnert et al. studied the impact of a low-salt vs. a normal and a high-salt diet on the CIA and K/BxN serum transfer-induced arthritis (STIA) [79]. In both mouse models, a low-salt diet significantly decreased arthritis severity, with less inflammatory joint infiltrates and cartilage breakdown. Moreover, IL-1 receptor blocking (in STIA) reduced complement-fixing anti-CII IgG2a levels and decreased anti-CII IgG2a/IgG1 ratios (as a more Th2-like response). In addition, reduced IL-17 and monocyte chemoattractant protein-1 levels (in CIA) were demonstrated.

The results of studies on the effect of a salt regimen in humans are less convincing. On the one hand, a low-salt (6 g/d for months) vs. a high-salt (12 g/d) diet in healthy individuals led to decrease in the blood monocyte number and reduced production of proinflammatory cytokines (IL-6 and IL-23), along with an enhanced ability to produce anti-inflammatory cytokine IL-10 [80]. On the other hand, no impact of a salt regimen on Treg/Th17 lymphocyte levels and in vitro Th17 cell differentiation was revealed in both healthy individuals and RA patients [81].

To further focus on RA, a comparison of synovial fluid between RA patients and OA patients revealed that Na^+^ and IL-17 were more abundant in RA synovial fluid, indicating a possible link of salt intake and rheumatoid inflammation [78]. Urinary Na/K ratio positively and significantly correlated with DAS28-ESR [82]. High daily sodium intake (estimated from foods plus added salt) in 18,555 individuals, including 392 self-reported rheumatoid arthritis individuals, showed a significant association with rheumatoid arthritis (OR = 1.5) [83]. In a nested case-control study, including 386 individuals who had stated their dietary median of 7.7 years before the onset of symptoms of RA and 1886 matched controls, revealed a link of high sodium intake with a more than doubled increased RA risk, but only among smokers [OR = 2.26) [84]. Jiang et al. revealed that high sodium chloride consumption enhances the effects of smoking in ACCP RA development [85].

Dynamic testing of RA in patients who were on a low-salt diet for 3 weeks (with analysis of urinary sodium excretion for confirmation adherence to the dietary regimen) and then returned to a normal salt diet revealed a trend toward a reduction in the Th17 cell frequencies and a countertrend for Treg and return to the previous levels after 2 weeks of following the normal salt regime with no significant apoptosis or altered proliferation [86]. It should be noted that publications on the problem of the link between RA and the salt regime are currently scarce. Apparently, given some encouraging findings, further research is needed for confirmation.

#### 2.4.1. Coffee

The results of the RA link with coffee consumption are ambiguous. On the one hand an increased risk was demonstrated (>4 cups) for RF-positive and ACCP-positive RA [87,88,89,90]. On the other hand, 1–8 cups per day appeared to have a protective effect [91]. At that level, multiple anti-inflammatory effects of caffeine are quite consistent with the protective effects of coffee: anti-inflammatory cytokines increased and pro-inflammatory cytokines decreased production, neutrophils and monocytes chemotaxis were inhibited, and B-cell antibody production decreased [92]. In addition, some coffee components possess antioxidant properties [93,94].

#### 2.4.2. Alcohol

On the one hand, moderate alcohol consumption reduces RA risk [66], but on the other hand, patients who had stopped drinking due to their illness or a desire to improve their health had worse physical functioning and higher levels in pain-related variables [95,96].

Studies of the mechanisms of alcohol consumption are quite consistent with the protective effect on RA development. Healthy premenopausal women having one drink/day had a significant serum increase in estradiol [97], with its remarkable anti-inflammatory effects. Alcohol consumption decreases systemic inflammation and inflammatory arthritis [98], diminishes response to immunogens and suppresses pro-inflammatory cytokine synthesis [99]. Alcohol addition to drinking water inhibits clinical signs of arthritis and joint destruction in mice and upregulation of testosterone production due to the decrease in NF-kB activation, cytokine/chemokine production and leukocyte chemotaxis [98].

#### 2.4.3. Smoking

The association of smoking and RA is also well-known. In a Swedish cohort of 277,777 male construction workers, chronic smoking was associated with increased RA risk (RR = 2.1) [100]. The pathophysiological mechanisms involve increased oxidative stress, apoptosis, development of a systemic proinflammatory state, autoantibody production, and interplay with genetic factors (Supplementary Table S2).

### 2.5. Burden of Society

#### 2.5.1. Residence

The incidence of RA in Taiwan cities is higher than in rural areas [101]. In the Polish cohorts, the physical condition of the sick urban citizens was more severe compared to the villagers [102]. Swedish studies did not reveal significant differences in RA incidence in sparsely populated areas and cities [103]. Conflicting results may be due to the ethnogenetic characteristics of cohorts, environmental differences, size of cities (in a Swedish study, a settlement with 25,000 inhabitants was considered a large city). The reasons for the heavier course of RA in cities may be exposure to traffic pollution, overcrowding, and more intensive contacts due to increased incidence of infections, the more intense rhythm of city life and provocation of mental stress.

#### 2.5.2. Mental Stress

Eustress is defined as favorable stress that invokes development of a balanced protective adaptive response (including Th1→Th2 shift of immune responses) to stressors in order to mobilize the body’s mental and physical reserves to resolve the situation [104]. This attests at least to the absence of any direct involvement of stress in provoking RA, that being a Th1-mediated disease. On the other hand, stress-induced shifts in the immune system may have serious consequences for susceptibility to infections [105,106]. A number of studies demonstrated the association of stress with acute respiratory rhinovirus infections, respiratory syncytial virus, Coronavirus 229E type, parvoviruses and herpesvirus infection reactivation [107,108]. All these viruses are known to be RA triggers [109,110,111]. However, in our opinion, only repetitive episodes of trivial infections are significant for provoking and maintaining RA activity. It is unlikely that a single infectious episode provoked by a normal anti-stress adaptive response in turn might provoke RA onset, unless that episode is the last straw in a series of immune system provocations of the genetically predisposed individual.

With excessive or chronic stress, or if there is a deviation in stress susceptibility, decompensated stress (distress) develops, and this is precisely the situation that triggers somatic disease development.

**Figure 2:**
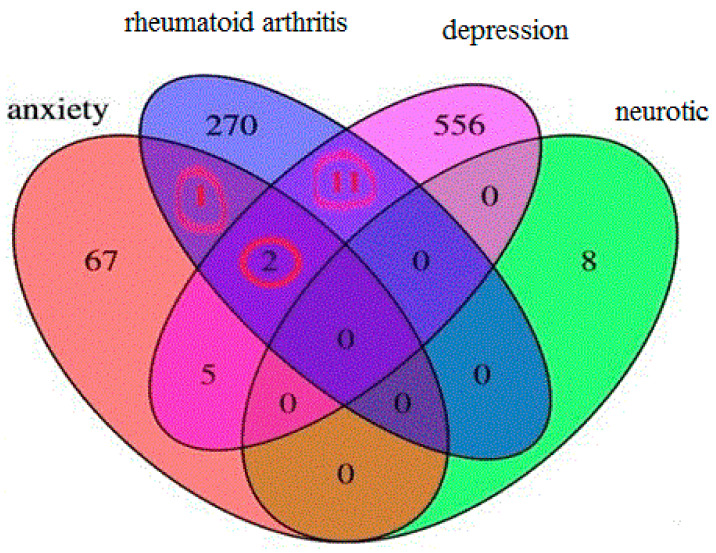
Search results in the GWAS Catalog: SNPs of 14 genes are associated with both RA and distress. The numbers of SNPs associated both with RA and distress are circled in red.

Analysis in the GWAS Catalog [112] revealed 14 matches of RA-and-depression-associated SNPs (Figure 2).

The comparison of RA and distress mechanisms indicates the possibility of mutual potentiation of these conditions (Supplementary Table S3) [113]. That is (1) typical for RA excessive NFkappaB pathway signaling and proinflammatory cytokine hyperproduction leading to the abolition or weakening of the Th1→Th2 shift in acute stress and promoting distress; (2) in RA, the features of HPA functioning with reduced production of glucocorticoids by the adrenal glands and, in some patients, a decrease in glucocorticoid receptor expression with the development of steroid resistance were demonstrated, leading to distress development via abolition or weakening of the Th1→Th2 immune shift in acute stress; (3) sympathetic/parasympathetic tone imbalance, detected in the preclinical RA stage, may contribute to distress development and (4) a reduced or even completely absent immune system cell response to catecholamines in RA may cancel or weaken theTh1→Th2 immune shift. Therefore, a detailed analysis reveals a certain synergy of triggering mechanisms of distress and RA. Minimal and unobtrusive stress for most individuals, for those with risk for RA, might provoke a prolonged pro-inflammatory cytokine production. Thus, the mental stress influence on RA development depends on the intensity of the stress, its duration, and individual characteristics of the HPA axis, which are obviously determined by genetic and epigenetic factors.

Hypothetical link of mental stress and RA:

Residence (urban/rural)
Psychological discomfort at work and at home—occupational hazards
⇓⇓⇓
Distress ⇒ infections ⇒ RA
⇓
NFkappaB signaling pathway and proinflammatory cytokine production ⇒ RA

#### 2.5.3. Sleep Deprivation

Regulation of the wake–sleep cycle is controlled by multiple neurochemical and molecular biological cascades, in which, in addition to the neuromodulatory system (in particular low molecular weight neurotransmitters and neuropeptides), other factors are involved, including HPA, the NFkappaB signaling system, cytokines, in regulating protein synthesis, protecting the brain tissue from oxidative or glutamatergic stress [114]. In connection with the problems discussed in this review, it is important to note that wake–sleep cycle fluctuations in HPA hormone levels (in particular, cortisol and cytokines) occur not only in the central nervous system but at the periphery as well. While the endocrine system was long believed to obey circadian rhythms and to be involved in sleep regulation, recently it has become evident that various immune system components also have a circadian rhythmicity. Therefore, both brain and peripheral cytokines are included in wake–sleep regulation, the most pro-inflammatory cytokines likely being somnogenic, whereas most anti-inflammatory cytokines are not. Peak activities of the pituitary hormones, prolactin and growth hormone production as well as another circadian pineal gland hormone—melatonin—and decrease in cortisol production occur overnight due to nocturnal sleep with the subsequent morning decrease in activity of nocturnal hormones and peak activity of cortisol after awakening [115,116]. All of these hormones are known to regulate immune system activity. Enhanced nocturnal prolactin, GH and melatonin concentrations as well as low cortisol levels and the following morning changes of the hormone activity are synergistically due to a Th1 shift at night and return to a Th1/Th2 balance of immune reactions in the morning [115,117]. These regularities were demonstrated not just for cytokine production, but in particular for the circadian rhythms of phagocytes and NK activities [114,117]. Normal circadian balance fluctuations might have a beneficial effect on the anti-infectious immune reactions. In humans the primary response to viral antigens following vaccination was enhanced by sleep [118,119,120]. A variety of disturbances in sleep duration and quality are due to an imbalance in the complex interactions of HPA hormones, melatonin and their receptors [116,117,118,119,120,121,122]. In particular, recurrent short sleep sessions were associated with a flatter diurnal cortisol pattern [121]. The disturbed circadian hormone modulation of the immune system leads to alterations in inflammatory gene expression [123] and upregulation of transcriptional pathways (e.g., NFkappaB) responsible for the inflammatory response [26], even in individuals in the absence of other health problems. So, sleep deprivation is associated with increased levels of the inflammatory markers C-RP [27,28]. Sleep deprivation may be due to the alteration of immune cell functions [114]: decrease in cell numbers of NK and other lymphocyte subsets, decrease in NK lytic activity, and decreased phagocytosis. Sleep deprivation might slow the catabolism of IgG. So, sleep disorders are fraught with a decrease in the effectiveness of anti-infective immunity. The proinflammatory shifts and disturbances in the HPA hormone activity caused by sleep deprivation bring these states closer to induction of mental stress and obesity. This triad is a fairly frequent combination [116,124,125]. Another important aspect is that short sleep duration and poor sleep efficiency in both mid and late pregnancy were associated with higher levels of IL-6 [126], and so, it might be a risk factor for adverse pregnancy outcomes [127]. In turn, besides the peculiarities of diurnal fluctuations of HPA hormones and melatonin, sleep disorders may be due to lifestyle factors and the aging process [128,129]. Therefore, sleep deprivation itself, as well as in connection with mental stress, obesity and with triggered adverse pregnancy outcomes, might play a role in provoking RA and its activity.

Sleep deprivation probability in persons under RA risk seems to be rather high. Various forms of clinically significant sleep disturbance were found in over 60% of RA patients [130,131]. Poor sleep quality correlated with greater pain severity, joint disability and RA activity [130,132,133,134]. High sleep deprivation incidence in RA is likely linked to the features of circadian ACTH, cortisol and prolactin, as well as melatonin level fluctuations [135,136,137,138], due to the demonstrated defect of HPA functioning.

Link of sleep deprivation and RA:

Sleep deprivation ⇔ Mental stress ⇔ Obesity ⇒ Proinflammatory shift ⇒ RA Sleep deprivation ⇔ Mental stress ⇒Increased susceptibility to infections ⇒ RA

Sleep deprivation ⇒ Increased risk of adverse pregnancy ⇒RA

### 2.6. Infections

Microorganisms and viruses are undoubtedly major RA triggers. Possible mechanisms triggering host autoimmune responses by pathogens are well known: molecular mimicry, epitope spreading, polyclonal lymphocyte activation, bystander activation and viral persistence [139,140,141,142]. All of these processes were demonstrated in RA [80]. The fact is that these mechanisms are beneficial phenomena, contributing to the immune system’s ability to attack multiple pathogens.

Therefore, the problem is not in these processes as such but is due to: (1) immune system disability to cope quickly enough with an infectious challenge due to RA-associated gene SNPs and abnormal downregulation of genes of some innate and adaptive immune system factors [110,143,144,145,146,147]; (2) imperfect control of the anti-infectious response, namely, inadequate lymphocyte activity modulation (PTPN22, CTLA-4,BTLA and other RA-associated gene SNPs), as well as an enrichment of RA-associated gene SNPs in NFkappaB and JAK/STAT signaling cascades due to the excessive proinflammatory response [14,148,149,150] and (3) the impaired sanitation of the infectious inflammatory focus from pathogenic and self-modified molecules due to pro-oxidant and antioxidant factor imbalance and inadequate activity of several enzymes involved in remodeling of the extracellular matrix [151,152,153]. Our long-term observations of RA patients and their first-degree relatives demonstrated that the persons under RA risk suffer from frequent and prolonged trivial infections. The peak of infections was observed within two years before RA onset and decreased in three years after RA onset. Nevertheless, infections continued playing a role in maintaining RA activity in the advanced stage of the disease. Increased incidence of excessive bacterial colonization in feces, urine, skin and nasopharynx samples of advanced RA patients without signs of infection indicates that the effectiveness of anti-infection resistance is relative, and a delicate balance may be disturbed. Both the whole set of infections carried over a year, and certain infections (purulent upper respiratory tract infections, acute and chronic tonsillitis exacerbations, skin infections and episodes of HSV infection reactivation) were demonstrated to actually be involved in RA triggering and persistence of RA activity [110,111,154].

Hypothetical link of infections and RA:

RA-associated HLA alleles ⇒ susceptibility to certain trivial infections ⇒ increased infection incidence and duration [155,156,157,158,159,160,161].

Technogenic burden (exotoxins, occupational hazards, mental stress, overcrowding) ⇒ infections;

Imbalanced anti-infection resistance ⇒ increased susceptibility to trivial infections ⇒ unbalanced anti-infection response (deficiency of some factors of innate immunity, prolonged pro-inflammatory cytokine production) ⇒ infectious pathogen persistence ⇒ lymphocyte inhibitory signaling systems deficiency⇒ impossibility of timely folding of the immune response;

Certain infections ⇒ posttranslational protein modifications in inflammatory sites ⇒ violation of readjustment inflammatory focus (imbalance of pro-oxidant and antioxidant factors, GST, MMP-3) ⇒ lymphocyte inhibitory signaling system deficiency ⇒ persistent RF, ACCP, anti-carbamylated protein antibodies (anti-CarP) production.

#### Occupational Hazards and Eco-Toxicants

The more severe RA in urban settings may be due to the more technogenic atmospheric emissions. RA incidence was inversely proportional to the distance of the residence from high-emission motorways [162,163]. Increased RA risk linked with silica, carbon monoxide, ozone, vapor, gas, dust and fume exposure, and cosmetic-associated mineral oil was demonstrated in a bulk of studies [164,165,166,167,168,169].

Another bulk of experiments revealed the impact of various ecotoxicants on basic intracellular processes, contributing to RA development (Supplementary Table S4). In particular, ecotoxicant-provoked oxidative stress might be important for moving a person at risk from one preclinical stage to another and to RA onset [170,171]. There are at least two mechanisms: (1) generated ROS stimulate activation NFkappaB signaling [172,173,174] and (2) oxidative stress can provoke protein carbamylation and the appearance of anti-Carp antibodies, intensively studied as RA prognostic markers [175,176].

**Figure 3:**
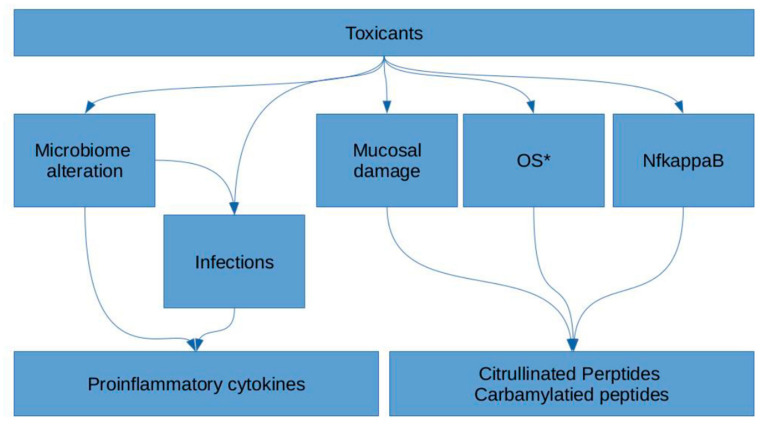
Possible mechanisms of implementation of triggering role of ecotoxicants in RA. OS*—oxidative stress.

The individual dispersive efficacy of ecotoxicant degradation mechanisms—a so-called “syndrome of nonspecific increased chemical susceptibility”, in particular, is manifested in immune disorders. The said dispersion might be due to the SNPs of detoxication system enzyme genes (Supplementary Table S5). At least three mutations were found to be RA-associated. Study of the function of the detoxication system can reveal an important link in the provocation of RA by non-genetic factors. It may turn out that ecotoxicant concentrations considered to be safe for the general population are fraught with RA provocation in persons at risk (Figure 3).

## 3. Concluding Remarks

**Figure 4:**
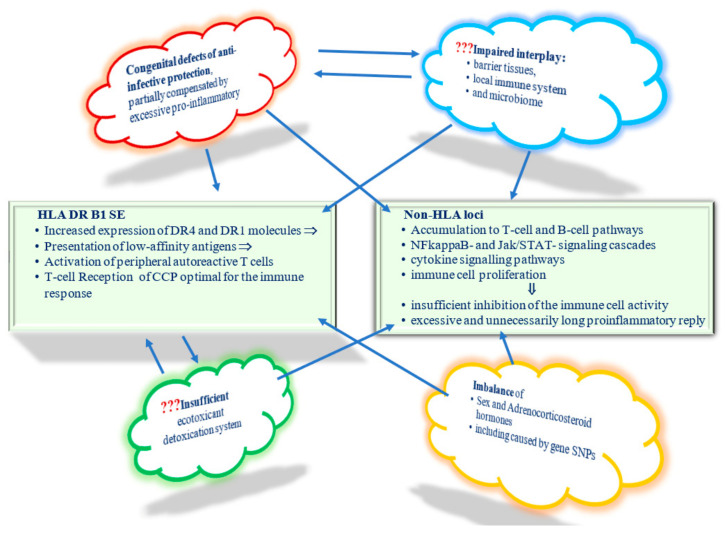
Mosaic of weak links of the body at RA risk.

The non-genetic factors modulate basic processes in the body (Figure 4), with the impact of these factors on the body being absolutely non-specific. The impact of these ordinary nonspecific factors—trivial infections, ecotoxicants in concentrations not exceeding the permissible values, such commonplace events as pregnancy, delivery, menopause—on the loci minoris resistentia of a body at risk of RA is to initiate an imbalanced protective adaptive response, which may provoke the disease onset. The most significant and well-known weak links are undoubtedly SE. The expression of these RA-associated variants of HLA DR B1 alleles is due to the presentation of low-affinity antigens, activation of autoreactive T lymphocytes and ACCP production. The other significant weak link is a poorly controlled and therefore beyond reasonable sufficiency pro-inflammatory response due to the bulk of SNPs accumulated in NFkappaB- and Jak/STAT, cytokine signaling pathways together with insufficient inhibitory control. It should be noted that RA is a phenotypically heterogenic pathology due to the sets of SNPs that differ from case to case. Therefore, hypothetically, there may be an RA subtype with congenital susceptibility to infections due to the SNPs of the genes involved in anti-infective protection and another RA subtype in persons with an imbalance in sex and corticosteroid hormones or an insufficient ecotoxicant detoxication system with matching sets of SNPs. Therefore, it can be assumed that one or another of the discussed effects may come to the fore in certain RA subtypes. Given the growing interest in the preclinical stages of RA, which are known to develop on the mucous membranes, perhaps the most promising line of research is to study the interplay of barrier tissues, the local immune system and the microbiome in persons at risk of RA.

**Figure 5:**
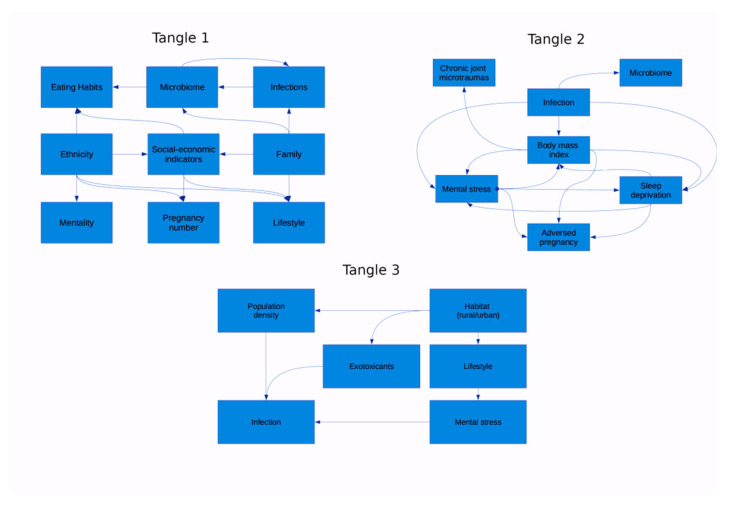
Network of challenging non-genetic factors.

It should be noted that environmental and individual factors affecting the loci minoris resistentia of a body at risk of RA form tangles of interdependencies, thus increasing their impact on the development of the disease (Figure 5).

**Figure 6:**
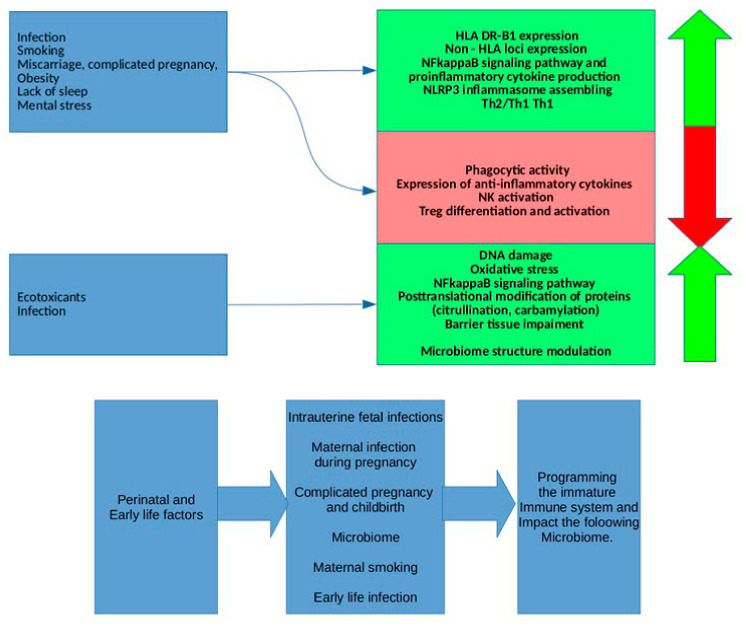
Impact of non-genetic factors on weak links of the body under RA risk.

Many non-genetic factors form the network of interdependent relationships; thus, several interdependent external factors can hit one weak body locus at once, either provoking or reinforcing each other (Figure 6).

Given the fact that the ratio of genetic and non-genetic RA risks is considered to be fifty/fifty, the algorithm for disease risk predicting should include both genetic and non-genetic factors, as well as any laboratory parameters indicating the negative impact of these factors. If we want to track down and, if possible, to prevent negative development of events at the earliest RA stages, the diagnosis of preclinical stages based on the presence of autoantibody/inflammatory markers is somewhat belated, not to mention that articular symptoms may be non-specific. This is not an easy task, keeping in mind the non-specific character of the parameters modified by external factors, the same as in the general population. It may be necessary to scale both the intensity of the external influence and the severity of response to it. The task is further complicated by the fact that the sets of gene SNPs that can lead to the development of RA can vary greatly from person to person. Indeed, RA is characterized by a wide variety of clinical manifestations (phenotypic heterogeneity).

That’s why a number of the more radical thinkers even believe that, in fact, the set of clinical signs that have been known to us since 1782 [177], and which we call "rheumatoid arthritis", is the outcome of many different pathogenic pathways—or, in other words, a syndrome resulting from a number of different diseases [52].

## Data Availability

The data discussed in the review were obtained in publications posted in PubMed.

## Supplementary Materials

The following supporting information can be downloaded at: https://www.mdpi.com/article/10.3390/ijms23158140/s1. References [6,22,52,109,144,178,179,180,181,182,183,184,185,186,187,188,189,190,191,192,193,194,195,196,197,198,199,200,201,202,203,204,205,206,207,208,209,210,211,212,213,214,215,216,217,218,219,220,221,222,223,224,225,226,227,228,229,230,231,232,233,234,235,236,237,238,239,240,241,242,243] are cited in the Supplementary Materials.
