# Supplementary material for: Interplay of Environmental, Individual and Genetic Factors in Rheumatoid Arthritis Provocation
Source: Int J Mol Sci. 2022 Jul 23;23(15):8140. doi: 10.3390/ijms23158140 (PMC9329780; doi:10.3390/ijms23158140)
Supplement: Supplementary file 1 [file ijms-23-08140-s001.zip › ijms-1784351-supplementary.pdf]

Table S1. Gender-associated events and rheumatoid arthritis

<table>
<tr><td colspan="2">Interplay of sex hormones and immune system</td><td colspan="2">Hormone dynamics in gender-associated events</td></tr>
<tr><td>Estrogens

shift Th1 ⇒ Th2

⇓ inhibits production and signaling of pro-inflammatory cytokines (TNF, IL1β and IL-6)

⇑ expression of anti-inflammatory cytokines (IL4, IL10, TGF)-β)

⇓ NK activation,

⇑Treg activation</td><td>Progesterone

switch Th1⇒Th2 phenotype

⇑ anti-inflammatory immune response

⇑Treg differentiation

⇓ NK</td><td>Normal pregnancy:

• extra levels of estrogens, progesterone, prolactin
• relatively small rise of testosterone levels
• shift to Th2 phenotype of immune activity [52]
Normal pregnancy, breast-feeding, hormone replacement therapy involve the activation of adrenocorticotropic hormone and cortisol production.</td><td>Delivery:

surge of Th1 shift and proinflammatory cytokine production.</td></tr>
<tr><td>Prolactin

⇑ antibody production

⇑ regulates CD4+ T cell maturation

⇑ pro-inflammatory cytokine production</td><td>Testosterone

⇓ pro-inflammatory cytokine production

⇑expression of anti-inflammatory cytokines

⇓Th1 differentiation

⇓Ig production and NK cytotoxic activity</td><td>Postpartum period

<u>without breastfeeding</u>

relatively rapid recovery to pre-pregnancy levels of estrogen, progesterone, testosterone</td><td>

<u>with breastfeeding</u>

some decrease in estrogen and progesterone levels (not to the levels before pregnancy)

high prolactin level (gradually decreasing throughout t 1 year).</td></tr>
<tr><td></td><td></td><td colspan="2">Adverse pregnancy:

reduced serum levels of estrogen and progesterone [179]

Th1/Th2 ratio – contradictory data [180]

Reduced cortisol levels in adversed pregnancy (pre-eclampsia, gestational hypertension) due to a lack of placental corticotropin-releasing hormone or reduced maternal ACTH [181]

Menopause

decrease of all hormone levels</td></tr>
<tr><td colspan="4">Presumptive link of gender-related events - hormone dynamics - rheumatoid arthritis risk</td></tr>
<tr><td colspan="2">Repeated normal pregnancies, childbirth, postpartum breastfeeding with normal feedback in the network of sex hormones and glucocorticoids ⇒bursts of production of these hormones with a protective effect ⇒ reducing RA risk;</td><td colspan="2">Normal pregnancy with hidden impairments of feedback in the network of sex hormones and glucocorticoids ⇒ RA onset within 1 year after delivery;</td></tr>
<tr><td colspan="2">Advers pregnancy as a clinical manifestation of impairment of feedback in the network of sex hormones and glucocorticoids ⇒RA triggering</td><td colspan="2">Menopause⇒decrease of hormone levels and their protective effects ⇒RA risk</td></tr>
</table>

Table S2. The known mechanisms of smoking impact, which may trigger RA

	Mechanisms
Smoking	Oxidative stress in the body altering mitochondrial membrane permeability [182].
	Activation of inducible NFkappaB [22].
	⇓⇑ apoptosis, depending on the cell types [184]
	Systemic proinflammatory state [185]
	Impaired antibacterial defense [185]
	Increase of number of alveolar macrophages and other monocytes in lung and levels of lysosomal enzymes and secreted elastase ⇒ parenchymal and connective tissue damage [186]
	Triggering of HLA-DR-restricted immune reactions to autoantigens modified by citrullination [187]
	Extensive genome-wide changes in DNA methylation [188]
	Gene-environment interaction between smoking and the HLA-DRB1 SE genotype [189]
	Gene-smoking interactions for glutathione S-transferase, N-acetyltransferase 2, mannose binding lectin genes [190]

a. The situation is aggravated in the presence of RA-associated SNPS of glutathione S-transferase T1 and mannose binding lectin genes [191,144].

Table S3. Synergism (⇑⇑) / antagonism (⇑⇓) of pathogenetic mechanisms of RA and Distress

Mechanism	Rheumatoid arthritis	Distress	⇑⇑/ or ⇑⇓
NFkappaB and proinflammatory cytokine	RA is characterized by accumulation of SNPs of the genes of NFkappaB signaling pathway as well as theJak/Stat signaling pathways due to excessive and poorly controlled production of proinflammatory cytokines [192,193]. Elevated levels of proinflammatory cytokines in the blood are detected in predisposing relatives of RA patients, which may be due to environmental factor provocation (e.g., infection) [109].It can be assumed that repeated acute mental stresses or chronic stress can also provoke a prolonged hyperproduction of pro-inflammatory cytokines in the individuals at risk of RA.	Acute stress leads to activation of the NFkappaB signaling pathway and tightly controlled short-term production of NFkappaB-associated proinflammatory cytokines in CNS and sera.Excessive and/or prolonged stress induced production of pro-inflammatory cytokines in the central nervous system and periphery is a risk factor for the development of chronic depressive disorder, anxiety disorders, aggression, and a number of neuropsychiatric diseases [194-196].	⇑⇑
HPA axis	A number of authors demonstrated defectsin neuroendocrine regulatory mechanisms, and a defective axis in the distal to the hypothalamus or pituitary gland in the HPA units. An alteration of the HPA stress response results in inappropriately low adrenal hormone secretions, cortisol in particular, in relation to ACTH secretion [197,198].A "relative adrenal insufficiency" – the reduced production of adrenal hormones related to the altered adrenal responsiveness to ACTH stimulation might impact RA onset, especially in elderly individuals, premenopausal women and in men [198-200].In addition, the reduced responsiveness of adrenal glands to ACTH stimulation may occur before clinical onset of the disease. In RA compared to healthy subjects, levels of ACTH, cortisol and other adrenal hormones were found to be markedly lower in relation to levels of IL6 and TNF. In that situation the number of swollen joints correlated inversely with the ratio of serum cortisol / IL6 [201].Besides the adrenal insufficiency, a number of the authors revealed pituitary-adrenal axis dysfunction in RA, i.e., a decrease of the CRH secretion by the hypothalamus [202].The analysis of data from 56 publications, led Geenen and coauthors to conclude that in response to mental and physical effort and applied physiological stressors, RA patients demonstrate a "too normal" HPA axis responsiveness considering the elevated immune activity [203].	Stress manifests itself by intense ACTH stimulated secretion of GK. Adequate activation of the axis by the stress factor with the production of a sufficient amount of GK is the most important condition for an antistress response that is harmonious in strength and duration. In particular, CRH and corticosteroids suppress the stress-inducing activity of the NFkappaB signaling pathway and the production of proinflammatory cytokines in the CNS and periphery on the principle of feedback. So, the adequate stress induced GK production might be due to the suppression of RA activity. Excessive and prolonged production of pro-inflammatory cytokines in the central nervous system and periphery in response to acute stress, which may be due to the inadequately low adrenal response in the individuals at risk for RA is a risk factor for the development of chronic depressive disorder, anxiety disorders, aggression, and a number of neuropsychiatric diseases [194,195].	⇑⇑

Corticosteroids and corticosteroid receptors[a]	In some RA patients the reduced levels of GK are combined with anomalies in the expression of glucocorticoid receptors. A significantly higher CRbeta mRNA expression level, and higherCRbeta/CRalpha ratio in the peripheral blood mononuclear cells were demonstrated in steroid resistant RA patients compared to steroid sensitive ones, and flow cytometry showed that the percentage of PBMCs staining for CRbeta protein was significantly higher in the steroid resistant RA group [204]. In addition to the possible dysregulation of the CRalpha function, alterations in the intracellular signaling mechanisms and/or utilization of various other cellular activation pathways, the inherited features of receptor functioning are assumed to be due to the SNPs inCRalpha and CRbeta genes, though the results are contradictory [205-207]. It is noteworthy that according to Chatzikyriakidou and coauthors (2009) the carriers of the N363 S and BclI minor alleles, associated with relative hypersensitivity to GCs, had a lower risk of developing RA and carriers of the 9β minor allele with relative GC resistance in contrast, had a higher risk of developing RA.	Insufficient production of corticosteroids within the framework of the anti-stress adaptive response and / or defects of glucocorticoid receptors in tissues, due to the steroid resistance are the major mechanisms for distress development	⇈
Sympathetic / parasympathetic balance	Analysis of 40 studies of sympathetic / parasympathetic balance in RA, performed byAdlan and coauthors revealed evidence of parasympathetic dysfunction in 77% and of sympathetic dysfunction in 53%of publications [208]. The authors of the majority of publications were unanimous in the conclusions on the reduction of parasympathetic tone in RA patients, as well as in persons at risk [209,210].Data on the sympathetic tone were contradictory [210-212].However, it should be noted that two studies revealed reduced catecholamine production in response to insulin-induced hypoglycemia as a stress factor in premenopausal women with low RA activity and persons with disease risk [213,214].In RA, the immunomodulatory function of catecholamines is likely abnormal. Wahle and coauthors revealed the abrogation of a catecholamine-induced Th1→Th2 shift in cytokine production by T-lymphocytes of RA patients [215]. These researchers also demonstrated that catecholamine-induced cell death of B lymphocytes is decreased in RA patients [216].A possible reason for the lack of immunomodulating effect is a decrease in receptors mediating the effect of catecholamines on immune cells [217].	Insufficient ejection of catecholamines and / or insufficient reduction in the tone of the parasympathetic nervous system, prolonged shift of balance towards sympathetic impulses in the central nervous system and periphery can lead to the development of depression [6].	⇈

Endogenous opioids	Most studies demonstrated a decrease of EO levels in sera, as well as in cerebrospinal fluid in RA [219-221].The beta-endorphin levels were also decreased in blood mononuclear cells [221].At that the EO levels had an inverse correlation with RA activity and severity both in sera and blood cells [219,222].Yin and coauthors demonstrated that clinical manifestations in rats with collagen-induced arthritis were significantly abrogated or ameliorated by treatment with beta-END [223].At the same time excessive levels of endorphin and enkephalin produced by leucocyte as well as the overexpression of mu-subtype opioid receptors on the various cells was demonstrated in rheumatoid synovial cells [224].It was demonstrated that EO may inhibit tumor necrosis factor-alpha and interleukin 1beta production at the level of mRNA expression, as well as MMP-9 production and its enzymatic activity by RA synovial cells [225]. EO treatment in vivo could down-regulate mRNA expression of several pro-inflammatory cytokines, chemokines and MMPs in CIA synovial, and polarize Th1/Th2 balance to Th2 [223].	Endogenous opioid hormones (EO) - endorphins, enkephalins and dynorphins, trigger a folding anti-stress adaptive response - inhibiting the activity of the stress system by a feedback mechanism.<br><br>Inadequate production of these factors is fraught with chronic stress	?

a.Corticosteroid receptor exists as alpha and beta isoforms. Only the alpha receptor binds corticosteroids. The CS/CRalpha complex binds to the glucocorticosteroid response element in the nucleus and also interferes with NF-kappaB binding, this mechanism being due to inhibiting of and NF-kappaB inducible genes, such as proinflammatory cytokines. CRbeta functions as an endogenous inhibitor of CS and is expressed in several tissues [226].

Table S4. Examples of the impact of some ecotoxicants on basic processes

Ecotoxicants	DNA damage	NFkB signaling pathway activation	Oxidative stress provoking
Heavy metals and their compounds			
Cadmium (Cd)	inhibition of DNA repair processes[51]	[227,228]	[229]
Mercury (Hg)		[229]	
Nickel (Ni)	[56]	[230,231]	[229]
Cobalt (Co)		[232]	[232]
Chromium (Cr)	DNA damage[57]	[233,234]	[229]
Copper (Cu)			[229]
Zinc (Zn)	DNA damage [59]	[228]	[235]
Arsenic (As)			[229]
Organiccompounds, gases			
Oxides of nitrogen		[236]	[237]
Carbon oxides		[237,238]	
Hydrocarbons		[239]	[240]

Freons			[240]
Silicon		[237]	[237]
Pesticides chlorinated		[241]	[242]
Nitrates		[243]	
Organic solvents (toluene, benzene, tetrachlorethylene)		[243]	

Table S5. Some known SNPs of detoxication system enzymes

Enzyme	Function	Known SNPs	Link with pathology	Author
Cytochrome 1A1	Enzyme of Phase 2 detoxification of xenobiotics<br>Transforms polycyclic aromatic hydrocarbons into carcinogenic products.	CYP1A1 I462V(1506 A-> G)<br>G allele increased activity of CYP1A1	An oncologic marker	
Microsomal epoxide hydrolaseEPHX1	Enzyme of Phase 2 detoxification of xenobiotics<br>Inactivation of toxic metabolites, which leads to the development of "oxidative stress	Tyr113His (T-337C)<br>T-337C is responsible for reducing the activity of the enzyme by 50% ("slow" allele)	Oxidative stress	[244]
Glutathione S-transferase (GSTP1)	Enzyme of Phase 2 detoxification of xenobiotics	GSTP1 I105V (A / G), A114V (C / T)<br>Combinations of A / B, B / V, B / C alleles with reduced	associated with an increased risk of cancer and multifactorial diseases of the female reproductive	

		activity	sphere (habitual miscarriage, infertility, complication of pregnancy	
Glutathione S-transferase (GSTM1)	Enzyme of Phase 2 detoxification of xenobiotics<br>Expression in liver and blood cells	GSTM1<br>3 allelic variants. GSTM1/A and GSTM1/B encode functionally adequate enzymes. GSTM1/0 - extended deletion (about 15 bp). 0/0 - lack active glutathione S-transferase. 0 / + - compensation for the absence of one active allele due to the full second. St. Petersburg - 38.8%, France - 44.6% Sweden and Great Britain - 42.0%, [71] Volga-Ural region 61.3 - 41.4%	Deletion polymorphism of GSTM1 increased RA susceptibility, particularly non HLA-DRB 1 SE carriers	[245]
Glutathione S-transferase (GSTT1)	Enzyme of Phase 2 detoxification of xenobiotics<br>Expression in liver and erythrocytes	GSTT1 -<br>0 allele - partial / complete deletion, protein is not synthesized. Dose-dependent effect: 0/0 absence of enzyme, +/- reduced enzyme activity ("slow conjugators"), + / + - normal glutathione	?	[246]

		transferase ability ("fast conjugators") The frequency of homozygous for the "zero" allele of GSTT1 genotype in populations of Caucasoids is 15-30%		
MDR1 3435	Transporter for many drugs, xenobiotics	C3435T allele	C3435T allele influence on the RA activity response to therapy	[246]
N-acetyltransferase 2	Involved in the metabolism of many xenobiotics	SNP are due to slow acetylation	NAT2 polymorphism – risk factor for joint destruction	[246]
